# Elaboration and Biocompatibility of an Eggshell-Derived Hydroxyapatite Material Modified with Si/PLGA for Bone Regeneration in Dentistry

**DOI:** 10.1155/2019/5949232

**Published:** 2019-12-05

**Authors:** Sandra Janeth Gutiérrez-Prieto, Luis F. Fonseca, Luis Gonzalo Sequeda-Castañeda, Kelly J. Díaz, Linet Y. Castañeda, José A. Leyva-Rojas, Juan Carlos Salcedo-Reyes, Adriana P. Acosta

**Affiliations:** ^1^Department of Dental Systems, Dentistry Research Centre, School of Dentistry, Pontificia Universidad Javeriana, Bogotá 110231, Colombia; ^2^Resident in Pathology and Oral Surgery, School of Dentistry, Pontificia Universidad Javeriana, Bogotá 110231, Colombia; ^3^Department of Chemistry, Faculty of Sciences, Pontificia Universidad Javeriana, Bogotá 110231, Colombia; ^4^Faculty of Sciences and Education, Universidad Distrital Francisco José de Caldas, Bogotá 110231, Colombia; ^5^Department of Physics, Faculty of Sciences, Pontificia Universidad Javeriana, Bogotá 110231, Colombia; ^6^Department of Periodontal Systems, Dentistry Research Centre, School of Dentistry, Pontificia Universidad Javeriana, Bogotá 110231, Colombia

## Abstract

Hydroxyapatite (HAp) is the most commonly used biomaterial in modern bone regeneration studies because of its chemical similarity to bone, biocompatibility with different polymers, osteoconductivity, low cost, and lack of immune response. However, to overcome the disadvantages of HAp, which include fragility and low mechanical strength, current studies typically focus on property modification through the addition of other materials. *Objective*. To develop and evaluate the biocompatibility of a HAp material extracted from eggshells and modified with silicon (Si) and poly(lactic-co-glycolic) acid (PLGA). *Materials and Methods*. An in vitro experimental study in which a HAp material prepared from eggshells was synthesized by wet chemical and conventional chemical precipitation. Subsequently, this material was reinforced with Si/PLGA using the freezing/lyophilization method, and then osteoblast cells were seeded on the experimental material (HAp/Si/PLGA). To analyse the biocompatibility of this composite material, scanning electron microscopy (SEM) and fluorescence confocal microscopy (FCM) techniques were used. PLGA, bovine bone/PLGA (BB/PLGA), and HAp/PLGA were used as controls. *Results*. A cellular viability of 96% was observed for the experimental HAp/Si/PLGA material as well as for the PLGA. The viability for the BB/PLGA material was 90%, and the viability for the HAp/PLGA was 86%. Cell adhesion was observed on the exterior surface of all materials. However, a continuous monolayer and the presence of filopodia were observed over both external and internal surface of the experimental materials. *Conclusions*. The HAp/Si/PLGA material is highly biocompatible with osteoblastic cells and can be considered promising for the construction of three-dimensional scaffolds for bone regeneration in dentistry.

## 1. Introduction

Every day, a significant number of people are affected by multiple procedures in the oral cavity, such as traumas, surgical resections, congenital defects, pathologies, and frequent chronic infections, including caries and periodontal disease that can lead to bone loss [1, 2]. Accordingly, the need for bone fillings and implants has increased in dental and medical clinical procedures, with bone grafting now one of the most frequently completed procedures each year [3]. Although the autograft is presented as the “gold standard” of clinical methods, due to its properties of biocompatibility, osteogenesis, osteoinduction, and osteoconduction, the autograft also possesses disadvantages such as morbidity of the donor site, the need for extensive surgeries, a high rate of reabsorption, and, above all, the limited availability of donor tissue, which has led some patients to forego treatment because of the scarcity of this tissue [3, 4]. To overcome these disadvantages, strategies have been developed in the field of regenerative medicine, including substitution with alloplastic materials, which although lacking osteogenic properties, have good mechanical properties in the presence of small defects, lower resorption rates, and a single surgical intervention, thus offering significant potential for clinical applicability [5–7].

One of the most commonly used materials is hydroxyapatite (HAp), which is extracted from various sources [8–13]. One potentially reliable and alternative source is eggshells, which are composed mainly of calcium and have shown good results in the regeneration of bone tissue [14–18]. HAp, due to its chemical similarity to bone, exhibits biocompatibility, bioactivity, and osteoconductive capacity, which allows its integration into bone tissue, in addition to being low cost [13, 14, 19–21]. However, HAp also presents low mechanical strength, minimal flexibility, and an unpredictable osseointegration. This is why other materials have been proposed in order to improve these disadvantages [22–30].

Numerous studies show silicon (Si) as an element that contributes to the formation of the bone and extracellular matrix, which in turn activates collagen production, a fundamental component of the bone matrix that when incorporated into the HAp, it resembles the mineral phase present in the bone and provides properties such as osteoconductivity, porosity, and bioactivity leading to improved osseointegration [30–33].

Likewise, other studies have shown that the (Si) added in different percentages on the structure of the HAp improves the adhesion, growth, and proliferation of osteoblast cells on its surface and also favours the formation of bone in the early stages, thanks to this element it increases the solubility of HAp by reducing the size of its grain. However, some studies also show that the solubility caused by Si on HAp alters its structure and mechanical properties, which must be optimized, in order to obtain a material that can be used as scaffolding in the engineering of tissues [34–41].

A good option with which a structured compound could be obtained is polylactic-glycolic acid (PLGA). This polymer has mechanical properties very similar to the bone such as flexibility, mechanical strength, versatility in its architecture, and biodegradability; the last one is a very important property in this material since it can affect cell viability, so it has been managed considering its proportions. Recent studies show that it also can be use as biodegradable microspheres for compound release. Another characteristic of this material is its hydrophobicity, which is why it has been mixed and modified with other compounds that allow the formation of collagen and makes it more hydrophilic and therefore more biocompatible [42–50].

In this study, a compound of HAp/Si/PLGA was manufactured and its biocompatibility was evaluated in order to be used as a possible scaffold with applicability in bone regeneration in dentistry. The HAp obtained from the eggshell was doped with Si and structurally characterized by scanning electron microscopy and energy-dispersive X-ray spectroscopy (SEM-EDS) and Fourier-transform infrared spectroscopy (FTIR) and analysed by Raman spectroscopy. Subsequently, to this material, PLGA was added through the freezing-lyophilization method and osteoblast cells were seeded, and their biocompatibility was evaluated in vitro through fluorescence confocal microscopy (FCM) and SEM.

## 2. Materials and Methods

### 2.1. Elaboration of the HAp Material from Eggshells

HAp material was synthesized from eggshells using chemical precipitation [51, 52]. After cleaning the eggshells, a high-efficiency mill was used to grind the eggshells and free them of impurities. Once the eggshell powder was obtained, a two-stage oven heat treatment was applied. In the first stage, the eggshell powders were heated at 450°C for 2 h with a heating rate of 5°C/min, eliminating the organic compounds and leaving only calcium carbonate [51–53]. In the second stage, the calcium carbonate was heated at 900°C for 2 h at a heating rate of 0.5°C/min, which released carbon dioxide (CO_2_) and yielded calcium oxide as the final product, as shown by the following reaction [51–53]:

(1) $$\begin{array}{c} \text{Eggshell }\overset{\Delta }{⟶}{\text{CaCO}}_{3}\overset{\Delta }{⟶}\text{ CaO}+{\text{CO}}_{2} \end{array}$$

As a highly hygroscopic compound, calcium oxide is readily hydrated by ambient moisture, resulting in calcium hydroxide (Ca(OH)_2_), as shown in the following reaction [53]:

(2) $$\begin{array}{c} \text{CaO}+{\text{H}}_{2}\text{O}\overset{\Delta }{⟶}\text{ Ca}{\left(\text{OH}\right)}_{2} \end{array}$$

To obtain a hydroxyapatite suspension, a suspension of calcium hydroxide Ca(OH)_2_ and a solution of KH_2_PO_4_ was prepared. The former suspension was added to the latter, dropwise, under conditions of mild heating and constant agitation. During this addition, the pH was maintained in a range of 10-11 by employing a solution of ammonia (NH_4_OH) to obtain a stoichiometric hydroxyapatite (Ca/P) = 1.67 [54, 55].

After complete addition, the reaction mixture was allowed to mature for 48 h at room temperature to remove any impurities. The precipitate was separated from the suspension by vacuum filtration and was then washed with distilled water until a pH of 8 was obtained. Subsequently, the filtered precipitate was oven-dried at 130°C for 6 h and was then pulverized with a mortar until a fine powder was obtained [54, 56].

(3) $$\begin{array}{c} 10\text{Ca}{\left(\text{OH}\right)}_{2}+6{\text{KH}}_{2}{\text{PO}}_{4}⟶{\text{Ca}}_{10}{\left({\text{PO}}_{4}\right)}_{6}{\left(\text{OH}\right)}_{2}+6\text{KOH}+12{\text{H}}_{2}\text{O} \end{array}$$

### 2.2. Doping Hydroxyapatite with Silicon (KH_2_PO_4_-TEOS)

The hydroxyapatite extracted from the eggshells was doped with silicon, for which, by means of an acid-base neutralization reaction, the appropriate quantities of the precursors were mixed as follows: 0.500 mol of Ca(OH)_2_ obtained from the eggshells, 0.0289 mol of dipotassium phosphate (KH_2_PO_4_), and 0.008 mol of tetraethyl orthosilicate (TEOS) (Sigma-Aldrich®), which was the source of the silicon.

**Figure 1:**
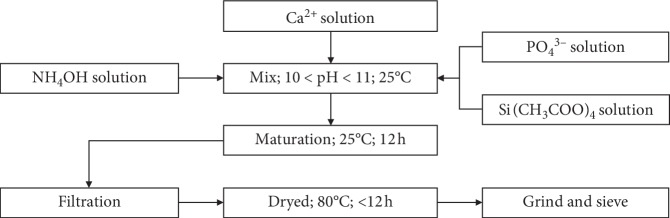
Flow diagram showing the synthesis of the HAp. The HAp material was prepared from the eggshell and synthesized by the conventional chemical precipitation method [54]. Afterwards, this was reinforced with Si/PLGA using the freezing/lyophilization method.

The precipitation reaction was carried out with constant heating and stirring, and the pH was maintained between 10 and 11 by the addition of ammonium hydroxide solution. After complete mixing of the precursors, the suspension was allowed to mature for 48 h and the resulting product was washed with distilled water, filtered, dried at 80°C for 6 h, and subsequently ground and sieved (Figure 1) [56–58].

### 2.3. Characterization of Hydroxyapatite Reinforced with Silicon (HAp/Si)

The HAp/Si material was characterized first by scanning electron microscopy and energy-dispersive X-ray spectroscopy (SEM-EDS) with a JSM-6490 LV of manufacture's house JEOL. Fourier-transform infrared spectroscopy (FTIR) was subsequently performed using Shimadzu Prestige-21. Finally, the material was analysed by Raman spectroscopy using an ID Raman Ocean Optics class 3B microscope [57, 58].

### 2.4. Addition of PLGA through the Freezing-Lyophilization Method

To add the PLGA to the material obtained from HAp/Si, 0.73 g of PLGA (Sigma-Aldrich®) and 8 ml of chloroform at 0.1% were mixed with 2.19 g of the HAp/Si powder in a ratio of 1 : 3, which was identified by considering the chemical composition of the bone. Once mixed in a 10 ml centrifuge tube (Falcon), the mixture was maintained for 24 h in a shaker to ensure complete dissolution of the polymer. Subsequently, the mixture was transferred to a beaker and mixed with a magnetic stirrer for 4 h, during which time 2 ml of distilled water was added dropwise. The obtained mixture was transferred to plastic moulds (Cryovial) and frozen for 24 h at −80°C. After this process, the material was compacted every 4 h for 2 days and subsequently lyophilized at −53°C for 24 h [59]. The samples were cut into disks with approximate dimensions of 6 mm (diameter) × 1.5 mm (thickness) and then sterilized with ethylene oxide [60].

### 2.5. Osteoblast Culture

Osteoblasts were acquired in vials of 500,000 cells from the third pass (3P) of human osteoblast cell line (from the Swiss company Lonza referenced as Clonetics™ Human Osteoblast Cell Systems). Each vial was thawed and then seeded in 2 culture flasks (S25), each containing 250,000 cells according to the protocol of the manufacturer. The culture medium used for these cells was OBM Clonetics™ supplemented with 5% foetal bovine serum (FBS), 0.1% insulin, 0.1% basic osteoblast growth factor, and 0.1% gentamicin/amphotericin B. Media changes were performed one day after seeding and every two days thereafter. The cell subculture was performed when, microscopically, a confluence between 70 and 80% was reached when using the Lonza Clonetics™ Human Osteoblast Cell Systems kit under a saline solution wash, trypsinization, and neutralization, followed by centrifugation at 2000 rpm for 5 min. The cells were subsequently resuspended in medium, seeded again in boxes of 25 cms, and allowed to expand until the fifth pass (5P).

### 2.6. Seeding of the Osteoblasts on the HAp/Si/PLGA Material

Hydroxyapatite disks made from eggshells mixed with silicon and PLGA (HAp/Si/PLGA) (experimental group) and the materials used as controls, which included PLGA, bovine bone/PLGA (BB/PLGA), and hydroxyapatite extracted from eggshells/PLGA (HAp/PLGA), were previously sterilized with ethylene oxide for 24 h and ultraviolet light for 1 h. Next, the disks were placed in boxes (well-transfer disks 35 mm in diameter) in triplicate, and subsequently, 1.5 × 10^5^ osteoblastic cells from the sixth pass (6P) were seeded on top of each material. At 8 days after seeding, the biocompatibility of each material was evaluated.

### 2.7. In Vitro Evaluation of the Biocompatibility of the HAp/Si/PLGA Material

#### 2.7.1. Analysis of Cell Viability

Cellular viability was assessed using fluorescence correlation spectroscopy (FCS) performed on Olympus FV1000 using a Live/Dead kit (Invitrogen, Carlsbad, CA USA) that uses calcein (excitation at 495 nm and emission at 515 nm), which discriminates living cells by green staining, and ethidium bromide (excitation 496 nm with emission at 635 nm), which discriminates dead cells by red staining. Five microliters of calcein and 20 *μ*l of ethidium homodimer were mixed in 10 ml of phosphate-buffered saline (PBS), following the instructions of the manufacturer. The culture medium of the cells in each box was removed, and 150 *μ*L of this solution was added directly to the cells on the material, allowing for 30 minutes of incubation before subsequent observation under a microscope. The percentages of the living and dead cells were estimated using ImageJ, which automatically counted the green and red cells present on the material using the following formula:

(4) $$\begin{array}{c} \text{Cell viability}=\frac{\text{live cells}}{\left(\text{live cells }+\text{ dead cells}\right)}\times 100. \end{array}$$

#### 2.7.2. Analysis of Cell Viability

Each of the matrices with cells was fixed with 2.5% glutaraldehyde for 4 h and subsequently dehydrated in increasing concentrations of ethanol (75%, 90%, and 100%) at 5, 10, and 20 minutes postfixation. The samples were passed through a critical point dryer (BALTEC CPD 030) and later were sputter-coated with a gold layer measuring 15 nm in thickness (BALTEC SCD 050). Once coated, the samples were observed under a scanning electron microscope (JEOL JSM-6490 LV) at an accelerating voltage of 15 kV, which was operated in the high-vacuum mode and equipped with a back-scattered electron detector.

## 3. Results

### 3.1. Characterization of the HAp/Si/PLGA Obtained

#### 3.1.1. Fourier-Transform Infrared Spectroscopy (FTIR)

**Figure 2:**
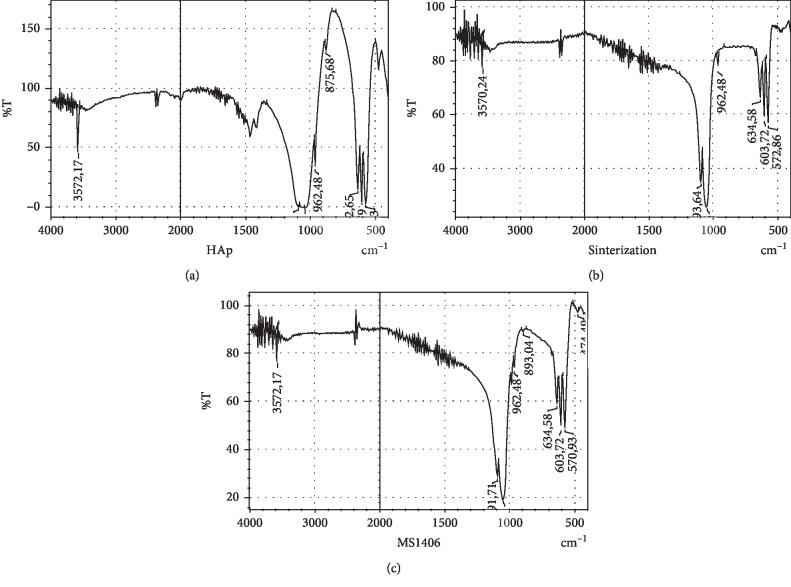
Spectra FTIR obtained from the hydroxyapatite material: bovine bone (a), eggshell (b), and eggshell doped with silicon (c).

Figure 2(a) shows the reference spectrum of the hydroxyapatite obtained from the bovine bone. In this spectrum, the bands identified at 3572 cm^−1^ and 632 cm^−1^ correspond to the stretching vibrations and structural flexion of hydroxyl groups (OH^1−^) [16, 19]. The 1035 cm^−1^ band shows the stretching of the P-O bond, while the bands at 1091 cm^−1^, 601 cm^−1^, and 570 cm^−1^ correspond to symmetric tension vibrations of the P-O bond. These assignments are confirmed by the band at 962 cm^−1^, which is characteristic of the stretching of the phosphate group (PO_4_^3−^) bonds [16, 17, 22]. The bands at 1458 cm^−1^ and 1421 cm^−1^ are characteristic of the flexion of the carbonate group (CO_3_^2−^) impurities, which occur as a product of the exchange of phosphates (PO_4_^3−^) by carbonates (CO_3_^2−^); at 873 cm^−1^, a band corresponding to a vibration in the form of flexion due to impurities such as hydrogen phosphate (HPO_4_^2−^) is observed [16, 17, 22].

Figure 2(b) shows the spectrum corresponding to the reaction between Ca(OH)_2_ and KH_2_PO_4_. The powders were sintered for further analysis. After sintering, bands are found at 3570 cm^−1^ and 634 cm^−1^, corresponding to stretching- and flexion-type vibrations of structural hydroxyl groups (OH^1−^) [13, 16], as well as bands at 1047 cm^−1^ corresponding to the stretching of the P-O bond, and at 1093 cm^−1^, 603 cm^−1^, and 572 cm^−1^ corresponding to symmetric tension vibrations of the P-O bond. These bands are consistent with the band that appears at 962 cm^−1^, which is characteristic of the stretching of the phosphate group (PO_4_^3−^) bonds [16, 17, 22].

Figure 2(c) shows the spectrum corresponding to Ca(OH)_2_, KH_2_PO_4_, and TEOS after the sintering process. In this spectrum, more defined bands are observed at 3572 cm^−1^ and 634 cm^−1^, both of which belong to the phosphate groups (PO_4_^3−^). At 1047 cm^−1^, the PO bond stretching is observed; at 1091 cm^−1^ and 603 cm^−1^, structural stretching and bending vibrations of the hydroxyl groups (OH^1−^) can be observed [16]. The band at 570 cm^−1^ corresponds to symmetric tension vibrations of the P-O bond, which is confirmed by the presence of the phosphate group (PO_4_^3−^) bond-stretching band at 962 cm^−1^ [16, 17, 22]. Likewise, stretching, bending, and rolling bands of the Si-O bond are visible at 673 cm^−1^ [14], and finally, the band at 474 cm^−1^ can be assigned to the groups corresponding to the presence of silicon (SiO_4_^−4^), which replace a fraction of the phosphate (PO_4_^3−^) and hydroxyl (OH^1−^) groups in the structure, the assignment of which is confirmed by the band appearing at 893 cm^−1^ [56].

#### 3.1.2. Raman Spectroscopy

**Figure 3:**
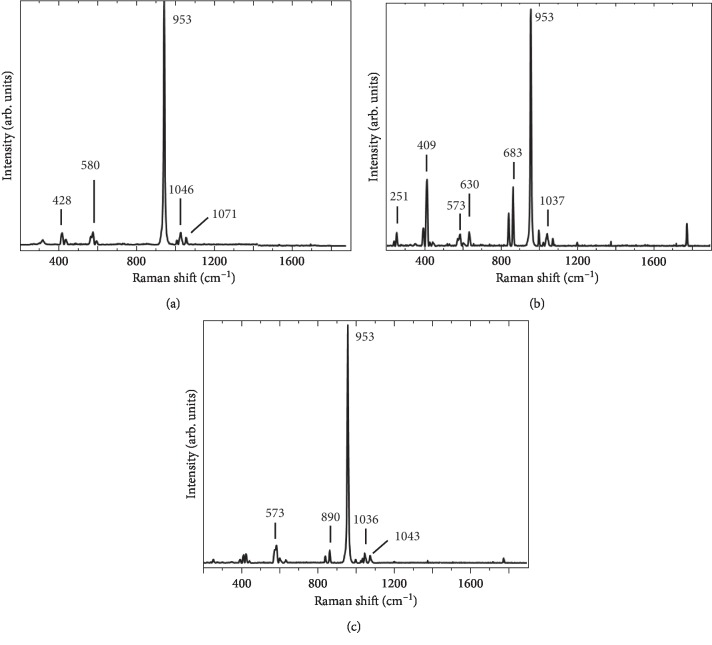
Spectra Raman obtained of hydroxyapatite material: bovine bone (a), eggshell (b), and eggshell doped with silicon (c).

Figure 3(a) shows the reference spectrum of hydroxyapatite obtained from the bovine bone, where the bands observed at 1035 cm^−1^, 1046 cm^−1^, and 1071 cm^−1^ correspond to the symmetric stretching of P-O [23, 24], as confirmed by the assignment of the band at 956 cm^−1^ [22, 28, 29]. The bands at 580 cm^−1^, 589 cm^−1^, and 607 cm^−1^ are assigned to the O-P-O bond vibrations [22, 28, 29]. In addition, the folding bands of the O-P-O bond are assigned at 470 cm^−1^ and 428 cm^−1^ [29].

Figure 3(b) presents the spectrum that shows the reaction between Ca(OH)_2_ and KH_2_PO_4_ (HAp) due to sintering, as evidenced by the band at 1037 cm^−1^, which corresponds to the symmetric stretching of P-O [23, 24, 26] and is confirmed by the assignment of the band at 953 cm^−1^ [24, 25, 29]. At 630 cm^−1^, the band corresponding to the release of hydroxide (OH^1−^) is observed [23], which does not appear in the reference spectrum, and at 573 cm^−1^ and 409 cm^−1^, bands corresponding to the O-P-O bond vibrations are assigned [24, 28].

Figure 3(c) presents the spectrum corresponding to Ca(OH)_2_, KH_2_PO_4_, and TEOS (HAp/Si) after sintering. The bands at 1043 cm^−1^ and 1036 cm^−1^ show the symmetric stretching of P-O [23, 24, 26], confirming the assignment of the band at 957 cm^−1^ [24, 25, 29]. The band at 573 cm^−1^ is assigned as an O-P-O bond vibration [24, 28]. In addition, the folding bands of the O-P-O bond [25] are assigned at 409 cm^−1^, and the band observed at 890 cm^−1^ is assigned to Si-O stretching [23].

### 3.2. Macrostructure of the HAp/Si/PLGA Material Obtained

**Figure 4:**
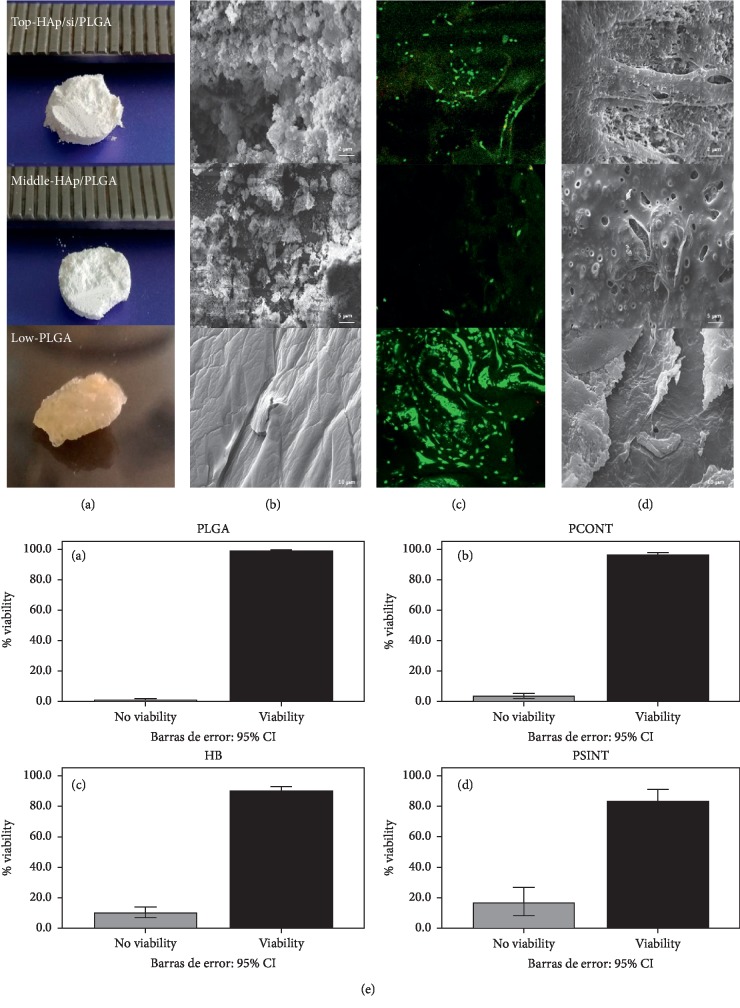
Structure and evaluation of the biocompatibility of the HAp/Si/PLGA material obtained, and its comparison with two of the control materials: HAp/PLGA and PLGA. Figure (e) Bar chart (ImageJ program) showing the viability percentage of the experimental material compared to the control materials. The highest percentage of live cells is observed in the PLGA (control, (a), and the HAp/Si/PLGA (b) (experimental) with a percentage of 96%, and the lowest percentage was presented by the HAp/PLGA (d) (control) with a percentage of 86%. The BB/PLGA shows a percentage of 90%.

Figure 4(a) (top) shows the macrostructure of the final Si-doped HAp material prepared by conventional chemical precipitation from eggshells and PLGA using freezing/lyophilization (HAp/Si/PLGA). The experimental material obtained was white and irregular in shape. Subsequently, this material was cut into disks measuring approximately 6 mm (diameter) × 1.5 mm (thickness). In contrast, the PLGA disk used as one of the control materials was more compact and browner (Figure 4(a) low) The most fragile material was HAp + PLGA.

### 3.3. SEM-EDS Evaluation of the Structure of the HAp/Si/PLGA Material

Figure 4(b) (top) shows the microstructure of the HAp/Si/PLGA material as observed using SEM at a magnification of 2500x. This image evidences an irregular, rough, and porous surface. In contrast, the PLGA control material exhibited a smoother surface, with several grooves and cracks (Figure 4(b) low). The EDS results confirmed the presence of silicon in the HAp/Si/PLG material at a concentration of 0.07%.

### 3.4. Evaluation of the Biocompatibility of the HAp/Si/PLGA Material Obtained

#### 3.4.1. Cell Viability by Fluorescence Correlation Spectroscopy (FCS)

Figure 4(c) (top) shows the image obtained by confocal fluorescence spectroscopy using the Live/Dead kit on osteoblast cells at day 8 of culture on the experimental material and on the control (Figure 4(c) Low). The percentage of cell viability was evaluated for all materials using ImageJ software and is shown in the bar graph in Figure 4(e).

#### 3.4.2. Evaluation of Cell Adhesion and Morphology by SEM

Figure 4(d) (top, middle, and low) shows the image obtained by SEM at day 8 of culture. A monolayer formation of osteoblast cells is observed on the external surface of all materials. The HAp/Si/PLGA material (Figure 4(d) top) shows on the external and internal surface filopodia characteristic of osteoblastic cells.

**Figure 5:**
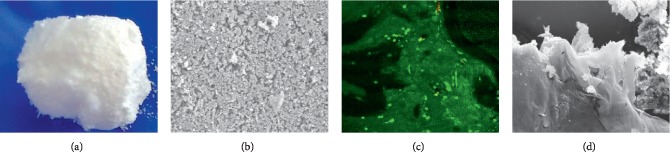
HAp/Si material: macrostructure (a) and microstructure (b) observed by SEM; cellular viability (c) observed by FCM and cellular adhesion and morphology (d) observed by SEM.

Furthermore, a HAp/Si control (Figure 5) was developed, as a point of comparison with the experimental material (HAp/Si/PLGA) in terms of its macro- and microstructure, and thus be able to observe the effect of Si on HAp without the addition of PLGA. In Figure 5(a), it is noted that the HAp/Si material presents great porosity, fragility, and a very whitish color. Microstructurally, it is observed that the grain size is small and oval and the architecture of the material is disordered and plain (Figure 5(b)). In contrast with the material to which the PLGA has been added (HAp/Si/PLGA), it is more compact, organized, less porous, and its color is slightly gray (Figure 4(a) top). Microscopically, it presents a larger grain size, less oval, more hexahedral, and organized with a moderated porosity (Figure 4(b) top). The HAp/Si material elaborated in this study upon contact with the culture medium for 8 days was dissolving until the material completely lost its initial architecture. However, cell viability can be seen through the FCM (Figure 5(c)), but SEM showed a lack in continuity of the monolayer due to the dissolution and fracture of the material (Figure 5(d)).

## 4. Discussion

To counteract the main disadvantages of HAp, such as its fragility and low mechanical strength, studies have focused on modifying its properties by forming HAp composites with different materials. Research shows that sintering of HAp at 1200°C increases its degree of crystallinity; however, when treated with Si, a submicron and microporous grain is created, which has a higher biaxial flexural strength than pure HAp but lower crystallinity [28].

The HAp material studied in the present work was derived from chicken eggshells because their membranes contain widely used essential nutrients such as calcium carbonate (CaCO_2_). Eggshells have typically been considered waste, with their usefulness in scientific applications underestimated. However, modern studies have focused on eggshells as a main component of HAp because they are extremely low cost, have unlimited availability, and are biocompatible and bioactive as a bone regeneration material [17].

The eggshell-derived HAp material has shown enhanced bone formation in comparison to synthetic hydroxyapatite [17]. In addition, this material is cheaper than synthetic HAp material [41] and can be manufactured from different precursors, such as K_2_HPO_4_, yielding nearly uniform sizes and regular shapes [22].

This material was doped with Si, and the methods used for analysis were SEM-EDS, FTIR spectroscopy, and Raman spectroscopy, which confirmed the presence of hydroxyapatite and substitution within the HAp structure by Si (Figures 2 and 3). It is known that the addition of Si to HAp improves the material's osteoconductivity and bioactivity due to the influence of Si on bone biochemistry, in addition to increasing the formation of new bone in vivo [27, 34] and the degree of mineralization [61].

The advantages associated with Si doping can be improved according to the proportions in which this material was added to the HAp in our study. Tsalsabila et al. [62] used the addition of Si proportions at 0.4%, 0.8%, and 1.2% by weight to hydroxyapatite, resulting in the successful substitution within the hydroxyapatite of Si at proportions of 0.4% and, especially, 0.8%. However, a decrease in the size of HAp crystals and a change in their shape were observed, showing more oval and less hexahedral crystals with no change in their surface [62].

In the present work, an average of 0.7% Si substitution by weight led to a more porous structure (Figure 4(e)) than that found with a fraction of 0.8%. At this amount, Si reduces the crystallinity of the structure and increases the solubility of the HAp powder [16]. In addition, the HAp particles imaged in this study exhibited the typical small oval shapes (Figure 4(e)) previously reported for HAp substitution with Si, e.g., Tsalsabila et al. [62] and Gibson et al. [63].

Other studies have indicated that controlling the equilibrium between the crystallinity and the biosolubility of the Si-HAp coating improves cell growth [41], a fact that was reflected in the present investigation, in which the fraction of added Si, along with the added fraction of PLGA, allowed for good adhesion of the osteoblastic cells seeded on the material, likely due to the increased porosity of the modified material reported here. A continuous monolayer of osteoblast cells adhered to the surface of the experimental material (HAp/Si/PLGA) was also observed, with the presence of filopodia characteristic of osteoblasts (Figure 4(d) top) and the observation of a large quantity of living cells inside the material, as revealed by FCS (Figure 4(c) top). The control material containing HAp/PLGA without Si exhibited a lower material porosity, the formation of a discontinuous monolayer, and a lower percentage of viable cells (Figures 4(c) and 4(d) middle); this suggests that the pores that the Si creates on the material and its interconnectivity are necessary for the cells to adhere and form an adequate monolayer, allowing it to survive. On the other hand, the HAp/Si material used as an additional control showed that in the absence of PLGA, it is a very fragile and rough material (Figures 5(a) and 5(b)) and in contact with the culture medium this material lost its architecture. However, it showed cell viability (Figure 5(c)) and formation of a monolayer, which showed breakage due to the fracture of the material that had been disintegrating (Figure 5(d)).

The biocompatibility results of the HAp samples combined with Si and PLGA (HAp/Si/PLGA) were consistent with those found by Gutierrez-Prieto et al. [19], in which the viability of osteoblast cells extracted from the knee was assessed for 4 materials (bovine bone, HAp, silicon, and collagen) by using SEM and fluorescence microscopy. Three of these materials were also used in the present study. Our results showed a high percentage of viability across the 4 material samples. A similar study was carried out by Parra et al. [64], in which the addition of Si and Mg to tricalcium phosphate significantly improved the viability and proliferation of the osteoblasts.

However, some studies have shown that in excessive amounts, the incorporated Si interferes with cell binding to the material because the Si significantly influences the nanocrystallinity of the Si-substituted HAp. As a result, the modified structure alters the interface between the bone cell and the biomaterial and induces bone cell apoptosis due to the loss of cellular anchoring [65, 66].

Thus, the addition of an adequate proportion of Si significantly improves the properties of the HAp material and gives the material greater bioactivity because it increases its protein adsorption capacity, improves adhesion and cell proliferation, and promotes greater control over the viscosity and stiffness of hydrogels, polymers, and ceramics. However, some in vivo studies have indicated that the combination of HAp and Si can occasionally generate an inflammatory response [67, 68].

PLGA was also added to the HAp in this study. This polymer has been shown to modify the biodegradability and interconnectivity properties of the HAp material and improve its cellular colonization. Likewise, this material neither seems to affect the ionic concentrations of Ca and P, nor the surrounding pH [59, 69]. The freezing-lyophilization method used to add PLGA in the present study was the same technique proposed by Raman [60], which was fast and efficient. However, it is important to manage the proportion of PLGA added with Si-doped HAp because depending on the concentration of HAp, the function of the seeded osteoblastic cells can either be stimulated or not. Therefore, in this study, it was decided to use a ratio of 1 : 3 by weight (0.73 g PLGA: 2.19 g HAp/Si), corresponding to a greater amount of HAp/Si with respect to PLGA.

On the other hand, the literature reports that bovine HAp combined with PLGA (BB/PLGA) tends to induce greater bioactivity than other forms of HA [9, 10]. The results of SEM and FCM in the present study suggest that the results of the BB/PLGA material, one of the materials used as a control (figure not shown) although it presented viability, were not as significant as that presented by the experimental material (HAp/Si/PLGA) and the PLGA control material. This is possibly because although HAp by itself helps cell adhesion for its porosity [63, 68], mixing it with Si and PLGA seems to increase its viability (Figures 4(c), 4(d), and 4(d) top).

In the present study, a HAp/Si/PLGA compound was obtained to be used as a scaffold allowing bone regeneration for its use in dentistry. For this material elaboration, properties reported in the literature for each of the elements were considered. The results obtained show that either macroscopically and microscopically, the material obtained has an irregular, porous, and rough structure, with relatively hexahedral shapes, typical of bone tissue (Figures 4(a) and 4(b) top) that allows the adhesion of osteoblastic cells on its surface (Figure 4(d) top SEM) and a cell viability of 96% (Figure 4(c) top FCM and Figure 4(e)).

These results also suggest that the proportions used of the 3 compounds (1 PLGA:3 HAp/Si) for their preparation, seem to be adequate since the porous material obtained showed very little disintegration in contact with the medium, practically retaining its initial architecture and showing a high cellular viability that it is apparently influenced by the proportions of the PLGA [45]. As for its biocompatibility, it could be that it is not only because of Si on HAp, but it could also have influenced Si on the surface of PLGA, making it more hydrophilic and allowing greater cell adhesion [43].

## 5. Conclusions

In this study, a HAp material was prepared from chicken eggshells modified with Si and PLGA polymer. Subsequently, these HAp samples were evaluated, with the results demonstrating that the resulting material is bioactive and biocompatible and can be used in the future for the construction of three-dimensional scaffolding, serving as a key element for bone regeneration. This material is also considered promising not only because of the characteristics studied here but also because of its self-sustainability, low cost, and future clinical applicability in the fields of dentistry and medicine. However, future studies with stem cells seeded on this material should be conducted to determine whether this material provides an adequate environment for these cells to differentiate. In addition, studies evaluating its biodegradability and mechanical properties are necessary.

## Data Availability

The data used to support the findings of this study are available from the corresponding author upon request.

## Supplementary Materials

Supplementary Materials The graphic abstract is included and is titled “Material HAp/Si/PLGA elaboration.”
