# Supplementary material for: Elaboration and Biocompatibility of an Eggshell-Derived Hydroxyapatite Material Modified with Si/PLGA for Bone Regeneration in Dentistry
Source: Int J Dent. 2019 Dec 5;2019:5949232. doi: 10.1155/2019/5949232 (PMC6915137; doi:10.1155/2019/5949232)
Supplement: Supplementary Materials — The graphic abstract is included and is titled “Material HAp/Si/PLGA elaboration.” [file 5949232.f1.pdf]

Graphical abtract of Material HAp/Si/PLGA elaboration: (A) Structural Material Characterization (SEM). (B) Evaluation of the biocompatibility of the HAp/Si/PLGA material. (C) Morphology and adhesión (SEM) of Osteoblastic cell seeded on experimental material. (D) Cellular Viability (CFS).

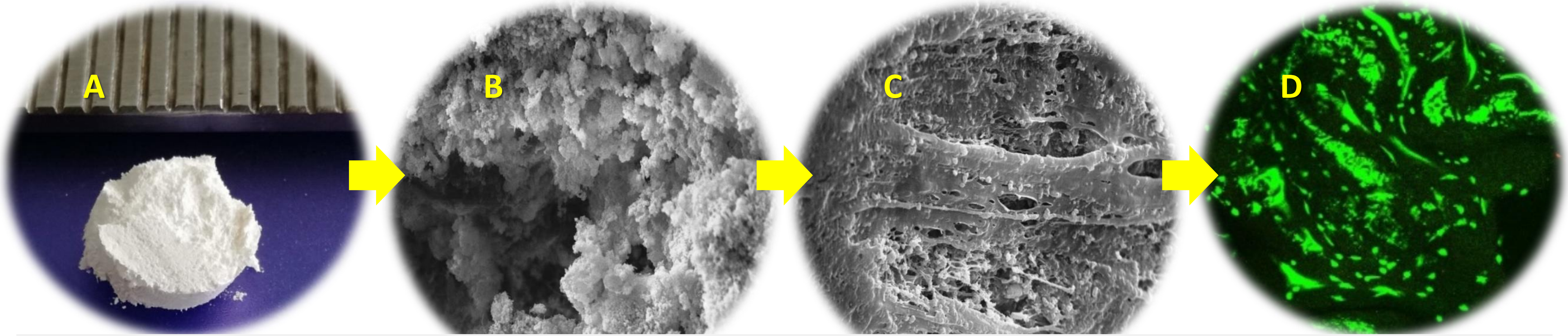

The graphic shows the elaboration and evaluation of the biocompatibility of a HAp material extracted from eggshells and modified with silicon (Si) and poly-lactic-co-glycolic acid (PLGA). With this objective, an in vitro experimental study was done in which a HAp material, prepared from eggshells was synthesized by wet chemical and conventional chemical precipitation. Subsequently, this material was reinforced with Si/PLGA using the freezing/lyophilization method, and then osteoblastic cells were seeded on the experimental material (HAp/Si/PLGA). Afterwards analysis of the biocompatibility of this composite material, was done through scanning electron microscopy (SEM) and fluorescence correlation spectroscopy (FCS) techniques. A cellular viability of 96% was observed for the experimental HAp/Si/PLGA material and Cell adhesion was observed on the exterior of its surface. A continuous monolayer and the presence of filopodia was observed also. These results suggest that the HAp/Si/PLGA material is highly biocompatible with osteoblastic cells and can be used for the construction of three-dimensional scaffolds with the purpose of bone regeneration in Dentistry.
